# Accelerated Wound Healing by Fibroblasts Differentiated from Human Embryonic Stem Cell-Derived Mesenchymal Stem Cells in a Pressure Ulcer Animal Model

**DOI:** 10.1155/2018/4789568

**Published:** 2018-12-30

**Authors:** Dajeong Yoon, Dogeon Yoon, Heejoong Sim, Inseok Hwang, Ji-Seon Lee, Wook Chun

**Affiliations:** ^1^Burn Institute, Hangang Sacred Heart Hospital, College of Medicine, Hallym University, Seoul, Republic of Korea; ^2^Department of Surgery, Hangang Sacred Heart Hospital, College of Medicine, Hallym University, Seoul, Republic of Korea

## Abstract

Fibroblasts synthesize and secrete dermal collagen, matrix proteins, growth factors, and cytokines. These characteristics of fibroblasts provide a potential way for fibroblast therapy to treat skin ulcers more effectively than conventional therapies such as cytokine therapy and negative pressure wound therapy. However, the obstacle to the commercialization of fibroblast therapy is the limited supply of cells with consistent quality. In this study, we tested whether human embryonic stem cell-derived mesenchymal stem cells (hESC-MSCs) could be differentiated into fibroblasts considering that they have characteristics of high differentiation rates, unlimited proliferation possibility from a single colony, and homogeneity. As a result, hESC-MSC-derived fibroblasts (hESC-MSC-Fbs) showed a significant increase in the expression of type I and III collagen, fibronectin, and fibroblast-specific protein-1 (FSP-1). Besides, vessel formation and wound healing were enhanced in hESC-MSC-Fb-treated skin tissues compared to PBS- or hESC-MSC-treated skin tissues, along with decreased IL-6 expression at 4 days after the formation of pressure ulcer wound in a mouse model. In view of the limited available cell sources for fibroblast therapy, hESC-MSC-Fbs show a promising potential as a commercial cell therapy source to treat skin ulcers.

## 1. Introduction

Skin injuries, such as burns, pressure ulcers, bruises, stab wounds, and abrasions, disrupt the skin barrier, resulting in infection, trauma, and scarring [1, 2]. Therefore, an adequate wound healing process including a complex interplay of immune and surrounding cutaneous cells is required. One major cell type involved in wound healing is dermal fibroblasts, which migrate into and proliferate at sites of injury in response to the release of growth factors such as epidermal growth factor (EGF), platelet-derived growth factor (PDGF), and transforming growth factor-*β*1 (TGF-*β*1) [3, 4]. Once at the site of injury, fibroblasts synthesize and deposit various cytokines and extracellular matrix-related macromolecules, including collagen and fibronectin (FN), to accelerate wound healing [5].

Generally, there are two types of skin wounds, acute wounds (e.g., stab wounds and burns) and chronic wounds (e.g., pressure ulcers, diabetic ulcers, and scars) [2, 3, 6]. The healing process of chronic wounds does not reflect the general processes of acute wound healing including hemostasis, inflammation, proliferation, epithelialization, and tissue remodeling [7, 8]. Therefore, chronic wounds are characterized by low proliferative capacity of fibroblasts and reduced growth factor levels or defects of a suitable protein matrix in the dermis [7, 8]. Accordingly, fibroblast therapy provides a possibility to compensate for defective fibroblasts in chronic skin wounds. Previous studies have suggested that fibroblast therapy is effective in treating recessive dystrophic epidermolysis bullosa (RDEB) [9–12] and skin ulcers [13, 14], whereas there was also a contradicting study indicating that human fibroblast-derived dermal substitute (Dermagraft) has shown little benefit for patients with venous leg ulcers [15].

Thus far, single cytokine therapy [16, 17], sequential cytokine therapy [18], negative pressure therapy [19], and fibroblast therapy [13, 14] have been applied to treat pressure ulcers. But above all, fibroblasts therapy has the highest potential to repair pressure ulcers compared to other therapies because fibroblasts synthesize and secrete human dermal collagen, matrix proteins, growth factors, and cytokines to create normal skin containing metabolically active, living cells [12]. In spite of these advantages, there are several hurdles for the commercialization of fibroblast therapy. First, another wound would be created if autologous fibroblasts were obtained at the normal site of a patient suffering from a pressure ulcer [20]. Second, fibroblasts are not appropriate cell sources for wound therapy due to their limited quantities and lifespan [21, 22]. Thus, if sufficient fibroblasts can be obtained through specific differentiation techniques, fibroblasts therapy may be commercialized, thereby allowing their recruitment into wound sites to promote wound healing.

Mesenchymal stem cells (MSCs) are characterized by easy isolation and expansion, safety, and differentiation potential into multilineage, homing effect, immunomodulatory function, and absence of ethical issue [23, 24]. Therefore, MSCs were widely used source of cell therapy in the field of regenerative medicine [23, 25]. Nevertheless, MSCs have limited cell numbers and replicative lifespan and different differentiation potentials dependent on individual [24, 26]. On the other hand, human embryonic stem cell-derived mesenchymal stem cells (hESC-MSCs) not only have all the advantages of MSCs but also can generate sufficient amounts of early passage MSCs with the consistent quality [27].

hESC-MSCs, established and investigated recently [27, 28], express typical MSC surface markers such as CD29, CD44, and CD90 and have the potential to differentiate into mesenchymal cells including adipocytes, osteocytes, and chondrocytes [27]. These cells have also been proven to be safe for therapeutic application through karyotyping and *in vivo* teratoma formation assay [27]. Furthermore, it has already been proven that hESC-MSCs showed high telomerase activity and therapeutic benefits in regenerative medicine [28–30]. Accordingly, hESC-MSCs might have the potential of fibroblast differentiation, and they could be unlimited cell sources of fibroblasts to overcome the drawbacks of currently existing treatments for pressure ulcers, considering that human MSCs can be differentiated into fibroblasts using connective tissue growth factor (CTGF; also known as CCN2) [31, 32].

In this study, we investigated the possibility of fibroblast differentiation using hESC-MSCs and tested the efficacy of hESC-MSC-derived fibroblasts (hESC-MSC-Fbs) along with hESC-MSCs in an *in vivo* mouse pressure ulcer model.

## 2. Materials and Methods

### 2.1. Reagents

Primary antibody against *β*-actin (sc-47778) was obtained from Santa Cruz Biotechnology (Santa Cruz, CA, USA). Primary antibodies against collagen type 1 (Col1; 234167) were obtained from Merck (Darmstadt, Germany). Primary antibodies against alpha-smooth muscle actin (*α*-SMA; a5228) were purchased from Sigma-Aldrich (St. Louis, MO, USA). Primary antibodies against VEGFA (ab46154) and FN (ab6328) were obtained from Abcam (Cambridge, UK). Primary antibody against CD31 (PECAM-1; TA313338) was obtained from OriGene (Rockville, MD, USA). RIPA buffer (R2002) was obtained from Biosesang (Seoul, South Korea). Protease and phosphatase inhibitor cocktail (11697498001) were purchased from Sigma-Aldrich (St. Louis, MO, USA).

### 2.2. Cell Culture and In Vitro Fibrotic Differentiation of hESC-MSCs

hESC-MSCs, kindly provided by Eun Ju Lee (Seoul National University Hospital, Republic of Korea), were cultured in microvascular endothelial cell media-2 (CC-3162, Lonza, Basel, Switzerland). For fibrotic differentiation, hESC-MSCs were seeded at 4.5 × 10^5^ cells/well (6-well plates). The medium was changed twice a week for 4 weeks and contained 2% fetal bovine serum, 50 *μ*g/mL ascorbic acid, and various concentrations of CTGF (10, 50, and 100 ng/mL). For animal experiments, hESC-MSC-Fbs differentiated from hESC-MSCs at 10 ng/mL CTGF were used.

### 2.3. RNA Isolation and Quantitative Real-Time Polymerase Chain Reaction (PCR) Analysis

Total RNA was isolated using Easy Blue reagent (Intron Biotechnology, South Korea) according to the manufacturer's protocol. Gene-specific primers are indicated in Table 1. All amplifications were conducted in a final reaction mixture (20 *μ*L) containing 500 nM gene-specific primers, 2x SYBR, and 6 *μ*L of template under the following conditions: denaturation at 95°C for 5 min, 40 cycles of 95°C for 10 s, 58°C for 15 s, and 72°C for 15 s and a final extension at 72°C for 5 min. Reactions were performed using a Roche LC480 instrument (Roche Diagnostics, Penzberg, Germany). Real-time PCR results were validated under the following conditions: denaturation at 95°C for 5 min; cycles (17 for *FN* and *β*-actin; 19 for *Col1*; 21 for *Col3*; 23 for *CD44*; and 25 for fibroblast-specific protein-1 [*FSP-1*]) of 95°C for 30 s, 58°C for 30 s, and 72°C for 30 s and a final extension at 72°C for 5 min using the same cDNA and primers. PCR products were separated on 2% agarose gels and visualized by ethidium bromide staining.

### 2.4. Masson's Trichrome Staining

Masson's trichrome staining (connective tissue stain) was performed according to the manufacturer's instructions (#SS1026-MAB-500, CANCER). Briefly, cryosection slides were placed in preheated Bouin's fluid for 60 min, followed by a 10 min cooling period. The slides were rinsed in tap water until sections were completely clear and then washed once in distilled water. The slides were then stained with equal volumes of Weigert's A and B for 5 min, and rinsed with running tap water for 2 min. Next, the slides were exposed to Biebrich scarlet-acid fuchsin solution for 15 min and rinsed with distilled water. The slides were differentiated in phosphomolybdic/phosphotungstic acid solution until collagen was no longer red and then rinsed with distilled water. Without further rinsing, the slides were treated with aniline blue solution for 5–10 min, followed by treatment with 1% acetic acid for 3–5 min and rapid dehydration with two changes of 95 and 100% ethanol. Finally, the slides were incubated with xylene and mounted with Balsam.

### 2.5. Immunoblotting

Cells were washed twice with ice-cold phosphate-buffered saline (PBS), lysed with an appropriate amount of tissue lysis buffer (RIPA buffer containing protease and phosphatase inhibitor cocktail), incubated on ice for 30 min, and centrifuged at 13,000 rpm for 10 min at 4°C. Next, 30 *μ*g of total protein was loaded and separated by sodium dodecyl sulfate polyacrylamide gel electrophoresis. Proteins were transferred to polyvinylidene difluoride membranes, blocked for 1 h with 5% nonfat dry milk in Tris-buffered saline (TBS) with 0.05% Tween-20 (TBS-T), and incubated with the appropriate primary antibodies in TBS containing 1% bovine serum albumin overnight at 4°C. Membranes were washed several times with TBS-T and incubated with horseradish peroxidase-conjugated secondary antibodies (0.1 *μ*g/mL; Jackson ImmunoResearch Laboratories, West Grove, PA, USA). Immunoreactivity was detected using an enhanced chemiluminescence detection system (WSE 6100 LuminoGraph I; ATTO, Tokyo, Japan).

### 2.6. Immunofluorescence Staining

Cells grown or differentiated on round glass coverslips in 24-well plates were fixed and permeabilized with 100% cold methanol for 10 min. Fixed cells were incubated for 1 h in PBS containing 3% bovine serum albumin for blocking, followed by 2 h of incubation with specific primary antibodies. Cells were washed three times with TBS-T, then incubated with Cy2-conjugated goat anti-rabbit/mouse IgGs (Jackson ImmunoResearch Laboratories) or Alexa 594-conjugated goat anti-rabbit/mouse IgGs (Molecular Probes, Eugene, OR, USA) as required according to the primary antibody. Cellular DNA was counterstained with 4′,6′-diamidino-2-phenylindole (0.2 *μ*g/mL in PBS).

### 2.7. Flow Cytometry Analysis

Cells (5 × 10^5^) were washed with PBS two times and stained with the following secondary antibodies conjugated with fluorophores: PE-29 (12-0299-41, Invitrogen), FITC-CD47 (11-0478-41, Invitrogen), PE-CD73 (550257, BD Biosciences), APC-CD90 (559869, BD Biosciences), PerCP-CD91 (46-0919-41, Invitrogen), PE-CD105 (560839, BD Pharmigen), and PE-CD166 (560903, BD Pharmigen) for 1 h. Information on the antibodies for the negative control is as follows: PE-IgG (555749, BD Pharmigen), APC-IgG (555751, BD Biosciences), PerCP-IgG (46-4714-82, Invitrogen), and FITC-IgG (11-4714-81, Invitrogen). The cells were washed with PBS two times and measured by flow cytometry on a FACSCalibur (BD Biosciences). The acquired data were analyzed using FlowJo software.

### 2.8. Wound Assessment

The length and width of each wound were assessed at the indicated times with a digimatic caliper (Mitutoyo, Sakado, Japan) to measure the length and width of each wound.

### 2.9. Immunohistochemistry

For immunohistochemical analysis, skin tissues were fixed with 10% formalin, soaked in 30% sucrose preservation solution, cryosectioned into 8 *μ*m thick sections, and stained with hematoxylin and eosin for the determination of the cell distribution. Additionally, sections were immunostained with anti-CD31 and anti-VEGFA antibodies and counterstained with hematoxylin for assessing angiogenesis.

### 2.10. *In Vitro* Cytokine Array

Expression of multiple inflammation-related cytokines was analyzed using the mouse inflammation antibody array C1 (AAM-INF-1-4, RayBiotech, GA, USA) followed by the manufacturer's instructions. Briefly, the array was performed with 300 *μ*g skin tissue lysates of each group (*n* = 3) at 4 days after treatment with the cells following pressure ulcer formation. For the quantification of dot images, cytokine levels in each membrane were calculated by computer-assisted image analysis using NIH ImageJ software (Bethesda, MD, USA). The relative expression levels in each group were determined by a simple algorithm offered from the manufacturer's protocol.

(1) $$\begin{array}{c} X\;\left(Ny\right)=\frac{X\left(y\right)*P1}{P\left(y\right)}, \end{array}$$

where P1 is the mean signal density (area) of positive control spots on the reference array (average of positive controls in the NT group), *P*(*y*) is the mean signal density (area) of positive control spots on array “*y*” (average of positive controls in PBS, hESC-MSC, or hESC-MSC-Fb group), and *X*(*y*) and *X*(Ny) are the normalized signal intensity (area) for spot “*X*” on array “*y*”.

### 2.11. Animal Experiments

#### 2.11.1. Maintenance of Mice

Male ICR mice (Hsd: CD-1, 25–30 g, 8 weeks old) were kept in the local animal care facility according to the institutional guidelines. Fifty-seven mice were included in these experiments: 3 mice in the normal group and 18 mice per treatment group (PBS-treated group, hESC-MSC-treated group, and hESC-MSC-Fb-treated group) in the pressure-ulcer model. Mice were caged separately in the animal laboratory under controlled conditions to optimize animal care. Mice had ad libitum access to rodent feed and water under standard laboratory conditions.

#### 2.11.2. Formation of Pressure Ulcers in Mouse Skin

For the formation of ulcers, pressure was applied to the shaved dorsal skin of mice using two opposite magnetic disks (about 1200 G magnetic force) for 12 h, which caused ischemia (I). The magnetic disks were then removed for 12 h, which caused reperfusion (R). These steps (I/R cycle) were repeated three times.

#### 2.11.3. Preparation of DiI-Stained Cells

Animal experiments through cell injection were done three times. Among them, animal experiments using DiI-stained cells were done two times, independently. hESC-MSCs and hESC-MSC-derived fibroblasts were trypsinized and neutralized with complete media. And then each cell was suspended with serum-free media at 1 × 10^6^ cells/mL, mixed with DiI dye (5 *μ*L/mL; cell labeling solution, V-22885, Invitrogen), and incubated for 5 min at 37°C. The labeled cells were centrifuged at 1500 rpm for 5 min. After that, the supernatant was removed, and the cells were gently resuspended in complete media. The wash procedure was repeated twice.

#### 2.11.4. Treatment of Pressure Ulcers with Cells

Immediately after the 3 I/R cycles, mice were separated into four groups and injected subcutaneously once in the wound margin as follows: normal control group, PBS-treated group, hESC-MSC-treated group (5 × 10^5^ cells/site), and hESC-MSC-derived fibroblast-treated group (5 × 10^5^ cells/site). This animal study was conducted in accordance with the guidelines and approval of the Institutional Animal Care and Use Committee of Hallym University (Hallym-2010-78).

### 2.12. Statistics

Graphical data are presented as the mean ± standard error of the means. Statistically significant differences among groups were determined using one- or two-way analysis of variance (ANOVA), followed by Bonferroni's post hoc, respectively.

## 3. Results

### 3.1. Fibroblast Differentiation of hESC-MSCs

**Figure 1:**
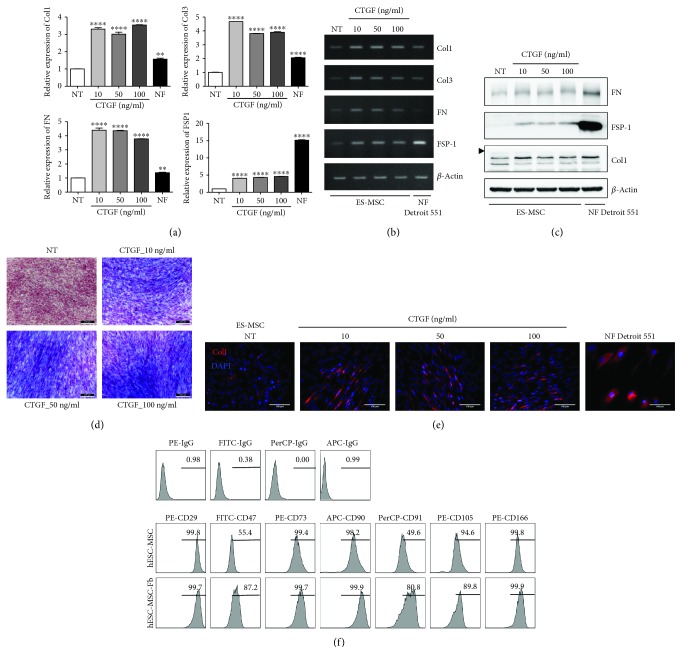
Fibrogenic differentiation of human embryonic stem cell-derived mesenchymal stem cells (hESC-MSCs) upon stimulation with connective tissue growth factor (CTGF). hESC-MSCs were differentiated into fibroblasts by treatment with various concentrations of connective tissue growth factor (CTGF) for 4 weeks. Normal skin fibroblasts (Detroit 551) were also used as a positive control. (a) mRNA levels of fibroblast-related genes in hESC-MSCs after CTGF treatment were determined by the real-time polymerase chain reaction (PCR) (*n* = 3, one-way ANOVA; ^∗∗^ *p* < 0.01 and ^∗∗∗∗^ *p* < 0.0001). (b) Collagen (Col)1, Col3, fibronectin (FN), and fibroblast-specific protein- (FSP-) 1 mRNA levels were determined by PCR. (c) FN, FSP-1, Col1, and *β*-actin protein levels in hESC-MSCs following CTGF treatment were determined by immunoblotting. (d) Masson's trichrome was used to detect collagen fibers. (e) hESC-MSCs were immunostained to detect collagen I (Col1) following CTGF treatment. 4′,6′-Diamidino-2-phenylindole (DAPI) was used for nuclear counterstaining. (f) Flow cytometry analysis of hESC-MSCs. After expansion of hESC-MSCs and hESC-MSC-Fbs, cells were trypsinized and stained with specific markers for CD29, CD47, CD73, CD90, CD91, CD105, and CD166.

Because bone marrow-derived MSCs can be differentiated into fibroblast-like cells [31, 32], hESC-MSCs were treated with fibroblast-inducing medium containing CTGF and ascorbic acid. Normal skin fibroblasts (Detroit 551) were also used as a positive control. Generally, primary fibroblasts expressed types I and III collagen (Col1 and Col3), fibronectin (FN), and fibroblast-specific protein 1 (FSP1) [31]. Therefore, the expression levels of Col1, Col3, FN, and FSP1 mRNA were evaluated after treatment of hESC-MSCs with 10, 50, and 100 ng/mL CTGF to confirm differentiation into the fibroblastic lineage. Along with morphological change, mRNA levels of Col1, Col3, FN1, and FSP1 were increased under conditions inducing fibroblast differentiation (Figures 1(a) and 1(b)). Similarly, Western blot analyses confirmed that the protein expression levels of fibrogenic markers, including FN, FSP-1, and Col1, were increased by treatment with CTGF (Figure 1(c)). Upon CTGF stimulation, hESC-MSC-Fbs were positive for Masson's trichrome (MT) staining compared with untreated hESC-MSCs (Figure 1(d)). Given the staining of collagen in blue and that of muscle fibers in red, hESC-MSCs were differentiated into fibroblasts. Moreover, Col1 was expressed in hESC-MSC-Fbs (Figure 1(e)). Thus, hESC-MSC-Fbs showed the ability to synthesize collagen. Furthermore, we performed flow cytometry to determine the expression of cell surface markers of hESC-MSC-Fbs (Figure 1(f)). The results showed that hESC-MSC-Fbs express more fibroblast-specific cell surface markers such as CD47 [33] and CD91 [34], compared to hESC-MSCs. In contrast, hESC-MSC-Fbs highly expressed cell surface markers expressed in both MSCs and fibroblasts, such as CD29, CD73, CD90, CD105, and CD166, similar to hESC-MSCs [35, 36].

### 3.2. Minimization of Skin Wound Size Induced by Pressure Ulcers Using hESC-MSC-Fbs

**Figure 2:**
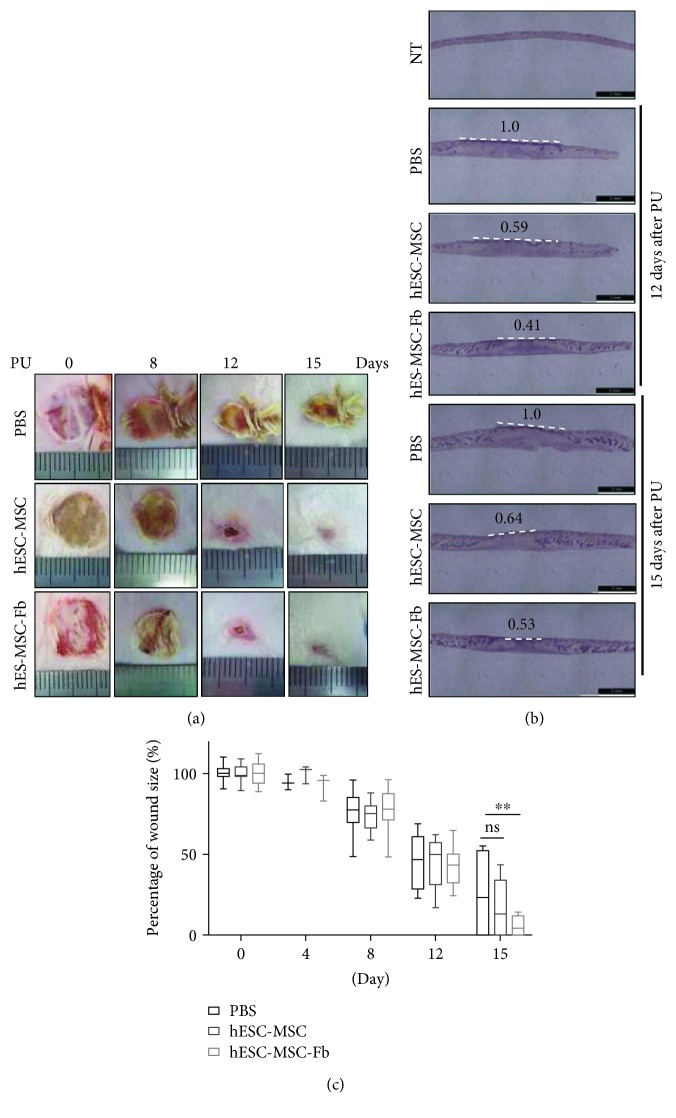
Differences in wound size following treatment with human embryonic stem cell-derived mesenchymal stem cells (hESC-MSCs) and hESC-MSC-derived fibroblasts (hESC-MSC-Fbs) after three ischemia/reperfusion (I/R) cycles. The dorsal skin of mice was subjected to three I/R cycles and injected subcutaneously with phosphate-buffered saline (PBS), hESC-MSCs, or hESC-MSC-Fbs. (a) Representative images of wounds after treatment are shown on the indicated days after three I/R cycles. (b) Hematoxylin and eosin staining of dorsal skin on 12 and 15 days after three I/R cycles. (c) Wound size was determined on the indicated days after treatment (*n* = 4, two-way ANOVA; ^∗∗^ *p* < 0.01).

Next, we used hESC-MSCs and hESC-MSC-Fbs to evaluate the efficacy of hESC-MSCs and hESC-MSC-Fbs as a source of cell therapy in a three I/R cycle-induced pressure ulcer (PU) mouse model. Treatment with hESC-MSCs and hESC-MSC-Fbs effectively reduced the size of pressure ulcer wounds over time (Figure 2(a)). In particular, the wound size in the hESC-MSC-Fb-treated group was reduced noticeably at 15 days after PU, compared to the PBS-treated group or hESC-MSC-treated group as indicated by hematoxylin and eosin staining (Figure 2(b)). Additionally, the size of the wound at different time points was measured using automatic calipers for each group (4 mice per group) and graphically represented for quantitative analysis (Figure 2(c)). The results indicated that only the group treated with hESC-MSC-Fbs showed a statistically significant reduction of wound size at 15 days after PU, leading to excellent healing of pressure ulcer-induced wounds.

### 3.3. hESC-MSC-Fbs as Alternative Dermal Constituents in Pressure Ulcer-Induced Skin Wounds

**Figure 3:**
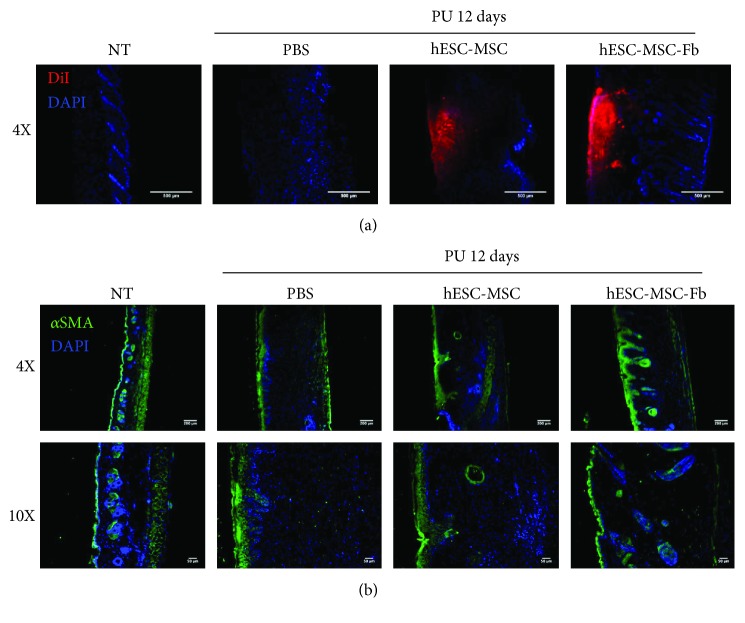
Arrangement of hESC-MSC-Fbs at the wound site after three ischemia/reperfusion (I/R) cycles. (a) Wounded skin after treatment with phosphate-buffered saline (PBS), human embryonic stem cell-derived mesenchymal stem cells (hESC-MSCs), or hESC-MSC-derived fibroblasts (hESC-MSC-Fbs) at 12 days after three I/R cycles was observed under fluorescent microscopy for the detection of DiI. (b) Wounded skin after treatment with PBS, hESC-MSCs, or hESC-MSC-Fbs at 12 days after three I/R cycles was immunostained for the detection of *α*-SMA. 4′,6′-Diamidino-2-phenylindole was used for nuclear counterstaining.

First, we wanted to identify the presence and location of the injected cells. Therefore, hESC-MSCs and hESC-MSC-Fbs were stained with DiI dye and then injected into wound margin after PU. Then, the remaining cells in the wound area were identified under fluorescence microscopy at the red wavelength to observe DiI fluorescence. Thus, we confirmed that hESC-MSCs and hESC-MSC-Fbs remained at the wound site at 12 days after PU (Figure 3(a)). Interestingly, DiI-positive cells within the injured skin were still visible at 4 weeks after injection of DiI-stained cells (data not shown). Next, skin samples were immunostained with *α*-SMA, which is mainly expressed in myofibroblasts and vascular smooth muscle cells, to visualize the arrangement of myofibroblasts within the wounded skin at 12 days after PU (Figure 3(b)). Notably, hESC-MSC-Fb-treated skin samples expressed *α*-SMA, similarly to a normal skin. However, the wound area in PBS- and hESC-MSC-treated skin samples did not heal properly, and the expression of *α*-SMA was minimal. Accordingly, these data imply that hESC-MSC-Fbs might be involved in the rearrangement of the skin injury site of pressure ulcers to improve wound healing.

### 3.4. Effective Healing of Pressure Ulcer-Induced Skin Wounds by hESC-MSC-Fbs

**Figure 4:**
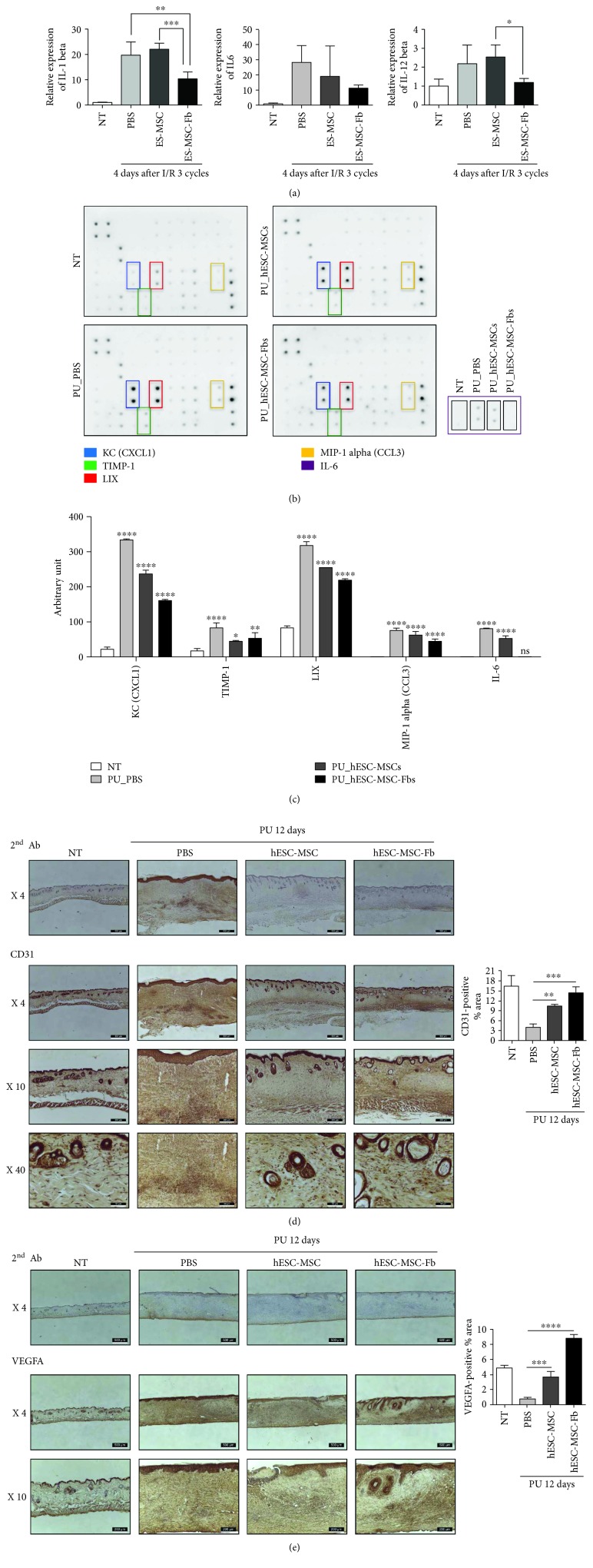
Effective wound healing after treatment with human embryonic stem cell-derived mesenchymal stem cell-derived fibroblasts (hESC-MSC-Fbs). (a) mRNA levels of inflammatory genes were determined in the wounded dorsal skin at 4 days after three I/R cycles by the real-time polymerase chain reaction (*n* = 4, one-way ANOVA; ^∗^ *p* < 0.05, ^∗∗^ *p* < 0.01, and ^∗∗∗∗^ *p* < 0.0001). (b) An *in vitro* cytokine array was performed on the wounded dorsal skin at 4 days after three I/R cycles. (c) The graph shows the quantified expression levels of several cytokines that showed differences between groups (*n* = 2, one-way ANOVA; ^∗^ *p* < 0.05, ^∗∗^ *p* < 0.01, ^∗∗∗^ *p* < 0.001, and ^∗∗∗∗^ *p* < 0.0001). (d, e) Wounded dorsal skin tissues at 12 days after three I/R cycles were immunostained for the detection of the angiogenesis markers CD31 (d) and VEGFA (e). Tissues were counterstained with hematoxylin. The graph shows the quantified expression levels of CD-31 (d) and VEGFA (e), relatively. Image quantification was performed through an ImageJ software program. (http://www.mecourse.com/landinig/software/cdeconv/cdeconv.html).

Furthermore, we tested the expression of early inflammatory genes of mouse skin tissue after cellular treatment following pressure ulcer formation because the reduction of initial inflammation is crucial to wound healing [3, 37]. Four days after the application of three I/R cycles, the mRNA expression of inflammatory genes including interleukin- (IL-) 1*β*, IL-6, and IL-12*β* was generally increased. Even though there is no statistical significance in mRNA expression of IL-6, the expression of inflammatory genes such as IL-1 *β*, IL-12*β*, and IL-6 was dramatically reduced in the injured skin tissue of hESC-MSC-Fb-treated mice (Figure 4(a)). Moreover, an inflammatory cytokine array was performed to check the expression of inflammatory cytokines in PU-induced skin tissue. Expression of inflammatory cytokines including KC (CXCL1), LIX, MIP-1 alpha, and IL-6 was significantly increased in PU-induced skin tissue (Figure 4(b)); the relative expression is shown in Figure 4(c). IL-6 levels were consistently decreased with regard to both mRNA regulation (Figure 4(a)) and protein synthesis (Figures 4(b) and 4(c)) in the injured skin tissue of hESC-MSC-Fb-treated mice. IL-6 is a cytokine involved in inflammation regulation and an important factor involved in wound healing [38, 39]. Decreased expression of IL-6 is closely related to scarless repair in fetal wound healing [40]. Therefore, we surmised that treatment with hESC-MSC-Fbs attenuated IL-6 expression in the early inflammatory response after the induction of pressure ulcers, leading to scarless wound healing.

Moreover, we performed CD-31 (PECAM-1) and VEGFA immunostaining to confirm that hESC-MSC-Fbs promoted angiogenesis to improve healing of skin wounds (Figures 4(d) and 4(e)). 12 days after the application of three I/R cycles, the formation of blood vessels was stimulated in the injured skin tissue treated with hESC-MSC-Fbs, but not PBS or hES-MSCs. Thus, these findings demonstrate that hESC-MSC-Fbs attenuated the inflammatory response at early time points during the development of pressure ulcers and promoted angiogenesis at later time points.

## 4. Discussion

As society ages, the number of patients with chronic wounds such as venous, arterial, pressure, and diabetic ulcers has increased [41–43]. Pressure ulcers, one of the typical chronic wounds, can be classified into four stages according to wound depth. Stage III and IV pressure ulcers encompass damage and necrosis of subcutaneous tissue, including dermis, by the loss of full-thickness skin [44, 45]. In general, pressure ulcer wounds are treated initially by debridement, wound cleansing, and dressing. In particular, additional treatment such as cytokine therapy [16, 18], negative pressure therapy [19], or fibroblast therapy [13, 14] would be beneficial in Stage III and IV pressure ulcer for the healing of the lost skin [44]. Therefore, the identification of an optimal therapeutic method to treat skin ulcers is essential.

Commercialization of fibroblast therapy would be enabled for the treatment of chronic wounds if the problems with current fibroblast therapies are solved. Recently developed hESC-MSCs have several advantages such as safety for teratoma formation, unaltered karyotype, proliferation potential from a single colony, homogeneity, and multidifferentiation potential [27, 28]. Previous studies also have shown that they have a healing effect in various animal disease models [27, 28, 30, 46]. There are other features that allow for hESC-MSCs to not be induced apoptosis upon treatment with substances that specifically kill human embryonic stem cells [47]. Accordingly, we would like to test whether hESC-MSCs and their differentiated fibroblasts have a wound-healing effect as a cell therapy source in the treatment of chronic wounds. We achieved fibroblast differentiation of hESC-MSCs and obtained a large quantity of fibroblasts derived from a single colony as a source of cells for therapy. After 4 weeks of obtaining fibroblasts, it was observed that distinctive properties of such fibroblast cells had not changed when they were frozen and thawed for the purpose of culture (data not shown). We applied hESC-MSCs and hESC-MSC-Fbs in a pressure ulcer animal model established through three I/R (12 h/12 h) cycles using magnetic disks [48, 49] and confirmed that hESC-MSC-Fbs lead to enhanced vessel formation and wound healing in pressure ulcer animal models. However, we think that it would have been a more ideal experimental design if the normal fibroblast was set for positive control during the experiment. In the future, we also would like to investigate whether the differentiated fibroblasts secrete several cytokines, considering that cytokine therapy is effective to treat pressure ulcers.

## 5. Conclusions

In conclusion, the results of this study demonstrate that hES-MSCs could be easily differentiated into fibroblasts under treatment with lower levels of CTGF than BM-MSCs [32]. Moreover, hESC-MSC- or hESC-MSC-Fb-treated wounds were smaller at 12 and 15 days compared to PBS-treated wounds. Additionally, injured skin tissues in the hESC-MSC-Fb-treated group showed the most effective wound healing based on the measurement of the wound size in mice within our experimental groups. Also, we found that hESC-MSC-Fbs were recruited into the wound site and acted to synthesize matrix proteins such as collagen and FN. In addition, injured skin tissues in hESC-MSC-Fb-treated mice showed reduced secretion of inflammatory cytokines (IL1 *β*, IL6, and IL12 *β*) at 4 days after three I/R cycles. We surmised that treatment with hESC-MSC-Fbs attenuated expression of IL6 at early time points after the induction of pressure ulcers, leading to scar-free wound healing considering that a previous study showed that scarless repair is closely related to the decreased expression of IL-6 in fetal wound healing [40]. In the future, it is considered necessary to confirm whether the increased myofibroblasts from the injured area are from the injected cells such as hESC-MSCs and hESC-MSC-Fbs or from peripheral normal fibroblasts. Taken together, our findings show that hESC-MSC-Fbs might have clinical applications as a source of cells for the treatment of pressure ulcers.

## Figures and Tables

**(a):** 

Role of genes	Genes	Origin	F/R	Primer sequences
MSC marker	CD90	Human	Forward	ATGAAGGTCCTCTACTTATCCGC
			Reverse	GCACTGTGACGTTCTGGGA
	CD44	Human	Forward	CTGCCGCTTTGCAGGTGTA
			Reverse	CATTGTGGGCAAGGTGCTATT
	CD29	Human	Forward	CAAGAGAGCTGAAGACTATCCCA
			Reverse	TGAAGTCCGAAGTAATCCTCCT

Fibroblastic hallmarks	Tn-C	Human	Forward	TCCCAGTGTTCGGTGGATCT
			Reverse	TTGATGCGATGTGTGAAGACA
	Col I	Human	Forward	GAGGGCCAAGACGAAGACATC
			Reverse	CAGATCACGTCATCGCACAAC
	Col III	Human	Forward	GCCAAATATGTGTCTGTGACTCA
			Reverse	GGGCGAGTAGGAGCAGTTG
	FN 1	Human	Forward	CGGTGGCTGTCAGTCAAAG
			Reverse	AAACCTCGGCTTCCTCCATAA
	FSP1	Human	Forward	GATGAGCAACTTGGACAGCAA
			Reverse	CTGGGCTGCTTATCTGGGAAG
	MMP-1	Human	Forward	GGGGCTTTGATGTACCCTAGC
			Reverse	TGTCACACGCTTTTGGGGTTT

Housekeeping gene	*β*-Actin	Human	Forward	TCCCTGGAGAAGAGCTACGA
			Reverse	AGCACTGTGTTGGCGTACAG

**(b):** 

Role of gene	Gene	Origin	F/R	Primer sequences (5′-3′)
Inflammation	IL-10	Mouse	Forward	CCAAGCCTTATCGGAAATGA
			Reverse	TTTTCACAGGGGAGAAATCG
	IL-6	Mouse	Forward	CCGGAGAGGAGACTTCACAG
			Reverse	CAGAATTGCCATTGCACAAC
	IL-1*β*	Mouse	Forward	TCCCAAGCAATACCCAAAGAAGAA
			Reverse	TGGGGAAGGCATTAGAAACAGTC
	IL-12*β*	Mouse	Forward	TGGTTTGCCATCGTTTTGCTG
			Reverse	ACAGGTGAGGTTCACTGTTTCT

Angiogenesis	PECAM-1	Mouse	Forward	ACGGTCTTGTCGCAGTATCA
			Reverse	TGGGTGCAGTTCCATTTTCG
	VCAM-1	Mouse	Forward	CAGCTAAATAATGGGGAACTG
			Reverse	GACGGTGTCTCCCTCTTTGA

Housekeeping gene	*β*-Actin	Mouse	Forward	AGTGTGACGTTGACATCCGT
			Reverse	TGCTAGGAGCCAGAGCAGTA

## Data Availability

All the data used to support the findings of this study are available from the corresponding author upon request.

## Abbreviations

hESC-MSCs: Human embryonic stem cell-derived mesenchymal stem cells
hESC-MSC-Fbs: Fibroblasts differentiated from human embryonic stem cell-derived mesenchymal stem cells
TGF-*β*1: Transforming growth factor-*β*1
FN: Fibronectin
RDEB: Recessive dystrophic epidermolysis bullosa
CTGF: Connective tissue growth factor
I/R cycles: Ischemia/reperfusion cycles
Col: Collagen
FSP1: Fibroblast-specific protein 1
*α*-SMA: Alpha-smooth muscle actin
IL: Interleukin
BM-MSCs: Bone marrow-derived mesenchymal stem cells.

## Supplementary Materials

Supplementary Materials Supplementary Figure 1: morphological images of fibroblast differentiation of human embryonic stem cell-derived mesenchymal stem cells (hESC-MSCs) upon stimulation with connective tissue growth factor (CTGF). Supplementary Figure 2: raw data of Figures 1(a), 2(c), 4(a), 4(c), 4(d), and 4(e). Supplementary Figure 3: images of CD31 staining taken from each group for statistical analysis.
