# Supplementary material for: Accelerated Wound Healing by Fibroblasts Differentiated from Human Embryonic Stem Cell-Derived Mesenchymal Stem Cells in a Pressure Ulcer Animal Model
Source: Stem Cells Int. 2018 Dec 30;2018:4789568. doi: 10.1155/2018/4789568 (PMC6332923; doi:10.1155/2018/4789568)

Supplementary Figure Legends

Supplementary Figure 1. Morphological images of fibroblast differentiation of human embryonic stem cell-derived mesenchymal stem cells (hESC-MSCs) upon stimulation with connective tissue growth factor (CTGF)

Supplementary Figure 2. Raw data of Figure 1A, 2C, 4A, 4C, 4D, 4E

Supplementary Figure 3. Images of CD31 staining taken from each group for statistical analysis

Supplementary Figure 1

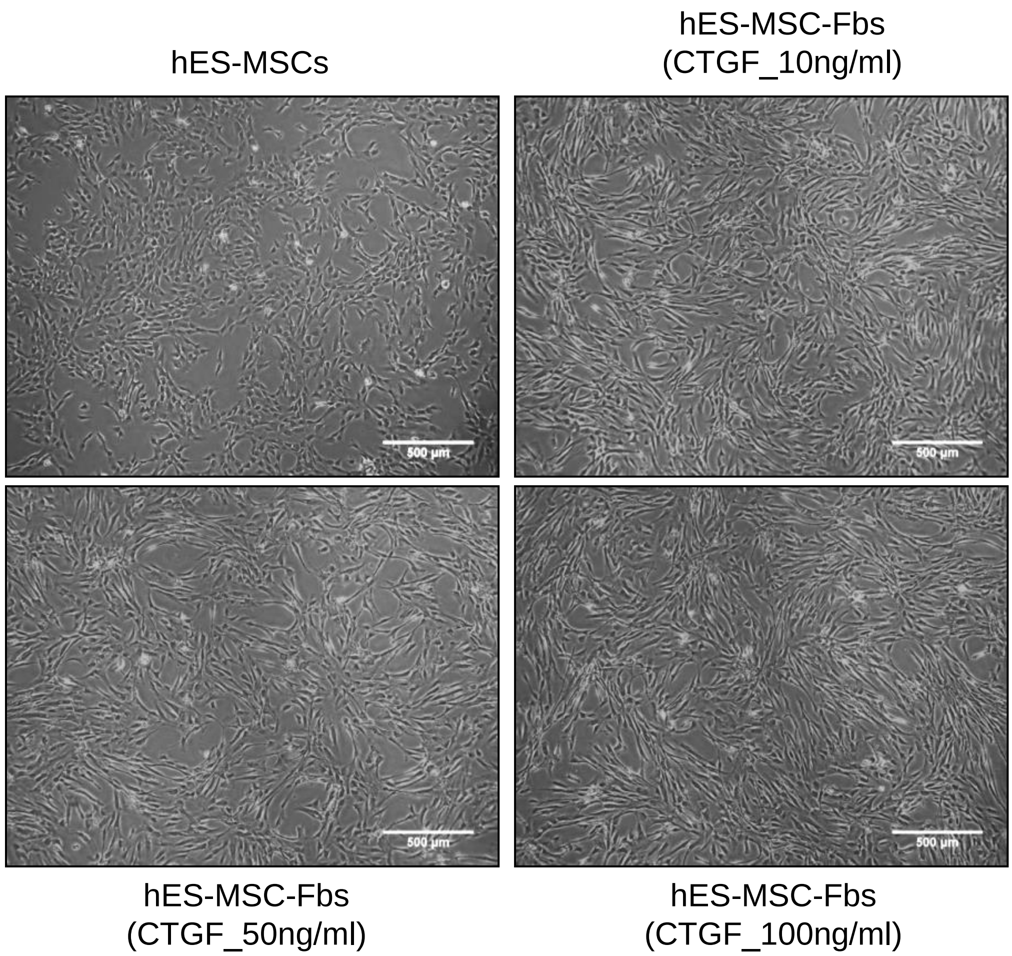

Supplementary Figure 2

Fig1A

a

Group	β-actin Ct	Col1 Ct	2^(Avg. β-actin Ct - Col1 Ct)	Average	Value
NT (1)	13.74	15.16	0.397768242	0.39915159	0.996534
NT (2)	13.92	15.15	0.400534939		1.003466
CTGF10ng/ml (1)	13.86	13.5	1.296839555		3.24899
CTGF10ng/ml (2)	13.89	13.45	1.342572503		3.363565
CTGF50ng/ml (1)	13.78	13.49	1.235418637		3.095111
CTGF50ng/ml (2)	13.81	13.57	1.168777249		2.928154
CTGF100ng/ml (1)	14.21	13.71	1.419123356		3.555349
CTGF100ng/ml (2)	14.22	13.72	1.409320755		3.530791
NF (1)	13.89	14.66	0.641712949		1.607692
NF (2)	14.15	14.73	0.611320139		1.531549

b

Group	β-actin Ct	Col3 Ct	2^(Avg. β-actin Ct – Col3 Ct)	Average	Value
NT (1)	13.74	18.7	0.034196678	0.034677378	0.986138
NT (2)	13.92	18.66	0.035158078		1.013862
CTGF10ng/ml (1)	13.86	16.5	0.162104944		4.67466
CTGF10ng/ml (2)	13.89	16.5	0.162104944		4.67466
CTGF50ng/ml (1)	13.78	16.71	0.132585968		3.823414
CTGF50ng/ml (2)	13.81	16.72	0.131670129		3.797004
CTGF100ng/ml (1)	14.21	17.09	0.136313467		3.930905
CTGF100ng/ml (2)	14.22	17.11	0.134436799		3.876787
NF (1)	13.89	17.85	0.070316155		2.027724
NF (2)	14.15	17.81	0.072293011		2.084731

c

Group	β-actin Ct	FN Ct	2^(Avg. β-actin Ct – FN Ct)	Average	Value
NT (1)	13.74	15.64	0.285190929	0.286182759	0.996534
NT (2)	13.92	15.63	0.287174589		1.003466
CTGF10ng/ml (1)	13.86	13.58	1.226884977		4.287068
CTGF10ng/ml (2)	13.89	13.51	1.28788163		4.500207
CTGF50ng/ml (1)	13.78	13.47	1.252664439		4.377149
CTGF50ng/ml (2)	13.81	13.48	1.244011653		4.346913
CTGF100ng/ml (1)	14.21	14.11	1.07549439		3.758068
CTGF100ng/ml (2)	14.22	14.1	1.082975046		3.784208
NF (1)	13.89	15.34	0.400534939		1.399577
NF (2)	14.15	15.39	0.386891248		1.351903

d

Group	β-actin Ct	FSP1 Ct	2^(Avg. β-actin Ct – FSP1 Ct)	Average	Value
NT (1)	13.74	22.48	0.002489376	0.002489376	1
NT (2)	13.92	22.48	0.002489376		1
CTGF10ng/ml (1)	13.86	20.49	0.01020203		4.098227
CTGF10ng/ml (2)	13.89	20.51	0.010061575		4.041806
CTGF50ng/ml (1)	13.78	20.33	0.010783729		4.3319
CTGF50ng/ml (2)	13.81	20.35	0.010635267		4.272262
CTGF100ng/ml (1)	14.21	20.66	0.01147788		4.610745
CTGF100ng/ml (2)	14.22	20.67	0.011398596		4.578897
NF (1)	13.89	18.74	0.03794359		15.24221
NF (2)	14.15	18.76	0.03742121		15.03236

Fig2C

		0day	4day	8day	12day	15day
PBS	1	1.81	9.79			
	2	8.88	8.82			
	3	9.65	9.25			
	4	9.65		8.3		
	5	9.78		7.37		
	6	9.87		6.26		
	7	9.78		8.3	6.77	
	8	9.52		8.49	6.26	
	9	10.12		9.16	5.82	
	10	10.17		7.61	3.74	
	11	10.12		7.6	2.66	
	12	9.82		6.83	4.69	
	13	9.86		8.39	4.3	
	14	8.88		6.89	2.85	0
	15	10.13		8.23	6.14	5.04
	16	10.14		9.42	4.59	3
	17	9.77		6.83	5.73	5.42
	18	9.58		7.28	2.76	1.58
	19	9.86		4.77	2.24	0
ES-MSC	20	9.78	10.36			
	21	9.85	10.19			
	22	9.38	9.32			
	23	10.85		7.48		
	24	9.85		6.17		
	25	9.77		8.47		
	26	8.9		7.26	3.56	
	27	9.78		7.99	6.15	
	28	10.51		7.77	5.12	
	29	10.15		7.61	2.86	
	30	9.53		7.91	5.55	
	31	9.8		6.17	1.69	0
	32	9.81		5.85	5.06	2.42
	33	10.42		7.03	6.18	1.3
	34	10.07		8.76	4.86	4.33
	35	10.64		7.37	3.19	0
ES-MSC_Fibro	36	8.71	9.7			
	37	9.2	8.14			
	38	9.85	9.39			
	39	10.01		8.52		
	40	9.45		4.75		
	41	10.53		9.44		
	42	9.24		7.63	4.25	
	43	9.82		7.66	3.17	
	44	10.65		8.82	6.36	
	45	9.41		7.01	3.46	
	46	8.75		6.62	3.47	
	47	11.02		8.33	2.38	0
	48	10.26		6.94	2.53	1.09
	49	9.8		7.63	4.36	0
	50	10.07		7.34	6.11	0.86
	51	9.02		7.8	4.93	0
	52	10.92		8.92	4.89	1.39

Fig4A

a

Group	β-actin Ct	IL1β Ct	2^(Avg. β-actin Ct - IL1β Ct)	Average	Group Average	Value
NT#1 (1)	19.78	26.6	0.009005	0.009037	0.010481	0.859219
NT#1 (2)	19.83	26.59	0.009068			0.865196
NT#2 (1)	18.7	25.13	0.011760	0.011925		1.12202
NT#2 (2)	18.74	25.09	0.012090			1.153564
PBS#1 (1)	16.04	18.27	0.219912	0.221447	0.206323	20.98227
PBS#1 (2)	16.13	18.25	0.222982			21.27517
PBS#2 (1)	16.12	19.02	0.140146	0.138704		13.37159
PBS#2 (2)	16.25	19.05	0.137262			13.0964
PBS#3 (1)	16.77	18.8	0.259715	0.258818		24.7799
PBS#3 (2)	16.94	18.81	0.257921			24.60874
ES-MSC#1 (1)	16.82	18.99	0.229251	0.231660	0.230975	21.87329
ES-MSC#1 (2)	16.91	18.96	0.234068			22.3329
ES-MSC#2 (1)	16.79	18.95	0.221442	0.205125		21.12821
ES-MSC#2 (2)	16.76	19.18	0.188809			18.01465
ES-MSC#3 (1)	16.49	19	0.256139	0.256139		24.43875
ES-MSC#3 (2)	17.58	19	0.256139			24.43875
ES-MSC-Fibro#1 (1)	16.25	19.17	0.134904	0.133515	0.108861	12.87141
ES-MSC-Fibro#1 (2)	16.31	19.2	0.132127			12.60652
ES-MSC-Fibro#2 (1)	17.91	21.53	0.083331	0.084207		7.950792
ES-MSC-Fibro#2 (2)	17.98	21.5	0.085082			8.117855

b

Group	β-actin Ct	IL6 Ct	2^(Avg. β-actin Ct – IL6 Ct)	Average	Group Average	Value
NT#1 (1)	19.78	30.22	0.000732	0.000658	0.000458	1.598839
NT#1 (2)	19.83	30.55	0.000583			1.271935
NT#2 (1)	18.7	30.8	0.000231	0.000259		0.504185
NT#2 (2)	18.74	30.49	0.000286			0.625042
PBS#1 (1)	16.04	21.76	0.019573	0.019438	0.012941	42.72547
PBS#1 (2)	16.13	21.78	0.019303			42.13726
PBS#2 (1)	16.12	22.79	0.010273	0.010566		22.42482
PBS#2 (2)	16.25	22.71	0.010859			23.70344
PBS#3 (1)	16.77	23.69	0.008759	0.00882		19.12018
PBS#3 (2)	16.94	23.67	0.008881			19.38709
ES-MSC#1 (1)	16.82	25.99	0.001791	0.001804	0.00872	3.909609
ES-MSC#1 (2)	16.91	25.97	0.001816			3.964186
ES-MSC#2 (1)	16.79	24.8	0.003839	0.003866		8.380431
ES-MSC#2 (2)	16.76	24.78	0.003893			8.497418
ES-MSC#3 (1)	16.49	22.7	0.019709	0.02049		43.02265
ES-MSC#3 (2)	17.58	22.59	0.021271			46.43126
ES-MSC-Fibro#1 (1)	16.25	24.12	0.004364	0.004349	0.005166	9.527017
ES-MSC-Fibro#1 (2)	16.31	24.13	0.004334			9.461209
ES-MSC-Fibro#2 (1)	17.91	25.33	0.005983	0.005983		13.05947
ES-MSC-Fibro#2 (2)	17.98	25.33	0.005983			13.05947

c

Group	β-actin Ct	IL12β Ct	2^(Avg. β-actin Ct - IL12β Ct)	Average	Group Average	Value
NT#1 (1)	19.78	35	0.000027	0.000041	0.000036	0.745899
NT#1 (2)	19.83	33.97	0.000054			1.523145
NT#2 (1)	18.7	33.51	0.000035	0.000031		0.987637
NT#2 (2)	18.74	33.92	0.000027			0.743319
PBS#1 (1)	16.04	30.62	0.000042	0.000045	7.78E-05	1.178583
PBS#1 (2)	16.13	30.42	0.000048			1.353836
PBS#2 (1)	16.12	30.11	0.000064	0.000067		1.798823
PBS#2 (2)	16.25	30.01	0.000069			1.92793
PBS#3 (1)	16.77	29.83	0.000124	0.000122		3.475096
PBS#3 (2)	16.94	29.89	0.000119			3.333535
ES-MSC#1 (1)	16.82	30.88	0.000060	0.000065	9.05E-05	1.690035
ES-MSC#1 (2)	16.91	30.68	0.000069			1.94134
ES-MSC#2 (1)	16.79	30.21	0.000090	0.000095		2.526347
ES-MSC#2 (2)	16.76	30.06	0.000100			2.803158
ES-MSC#3 (1)	16.49	30.01	0.000124	0.000112		3.475096
ES-MSC#3 (2)	17.58	30.34	0.000099			2.764566
ES-MSC-Fibro#1 (1)	16.25	30.77	0.000043	0.000044	4.27E-05	1.215924
ES-MSC-Fibro#1 (2)	16.31	30.74	0.000044			1.241473
ES-MSC-Fibro#2 (1)	17.91	32.22	0.000050	0.000041		1.411328
ES-MSC-Fibro#2 (2)	17.98	32.86	0.000032			0.905667

Fig4C

a

	NT	PBS	POS(NT)/ POS(PBS)*PBS	ES-MSC	POS(NT)/ POS(ES)*ES	ES-MSC-Fib	POS(NT)/ POS(Fib)*Fib
**KC (CXCL1)**	17	238	335.5300699	148	228.809816	151	157.8921162
	26	235	331.3006993	158	244.2699387	156	163.120332
**TIMP-1**	12	66	93.04615385	28	43.28834356	62	64.82987552
	22	52	73.30909091	30	46.3803681	40	41.82572614
**LIX**	87	231	325.6615385	165	255.0920245	212	221.6763485
	79	220	310.1538462	165	255.0920245	207	216.4481328
**MIP-1 alpha (CCL3)**	-	57	80.35804196	45	69.57055215	47	49.14522822
	-	50	70.48951049	36	55.65644172	39	40.78008299
**Positive average(POS)**	168	119.1666667		108.6666667		160.6666667	
		POS(NT)/ POS(PBS)		POS(NT)/ POS(ES)		POS(NT)/ POS(Fib)	
		1.40979021		1.54601227		1.045643154	

b

	NT	PBS	POS(NT)/ POS(PBS)*PBS	ES-MSC	POS(NT)/ POS(PBS)*ES	ES-MSC-Fib	POS(NT)/ POS(PBS)*Fib
**IL-6**	-	0.001	0.001548352	0.0008111	0.000905385	-	-
	-	0.0009778	0.001513979	0.0009778	0.001091463	-	-
**Positive average (POS)**	0.000889	0.000574		0.000796		0.000470	
		POS(NT)/ POS(PBS)		POS(NT)/ POS(ES)		POS(NT)/ POS(Fib)	
		1.548352446		1.116243538		1.889766849	

Fig4D

	Group	Area(%)	Average
CD31	NT(1)	15.718	16.383
	NT(2)	19.662	
	NT(3)	17.929	
	NT(4)	12.223	
	PBS(1)	4.675	3.8753
	PBS(2)	4.814	
	PBS(3)	3.788	
	PBS(4)	2.224	
	ES-MSC(1)	10.318	10.5405
	ES-MSC(2)	11.003	
	ES-MSC(3)	10.055	
	ES-MSC(4)	10.786	
	ES-MSC-Fibro(1)	15.4	14.4075
	ES-MSC-Fibro(2)	12.796	
	ES-MSC-Fibro(3)	16.569	
	ES-MSC-Fibro(4)	12.865	

Fig4E

	Group	Area(%)	Average
VEGFA	NT(1)	4.51	4.89
	NT(2)	5.18	
	NT(3)	4.98	
	PBS(1)	1	0.7967
	PBS(2)	0.55	
	PBS(3)	0.84	
	ES-MSC(1)	4.51	3.6967
	ES-MSC(2)	3.44	
	ES-MSC(3)	3.14	
	ES-MSC-Fibro(1)	8.27	8.7667
	ES-MSC-Fibro(2)	9.37	
	ES-MSC-Fibro(3)	8.66	

Supplementary Figure 3

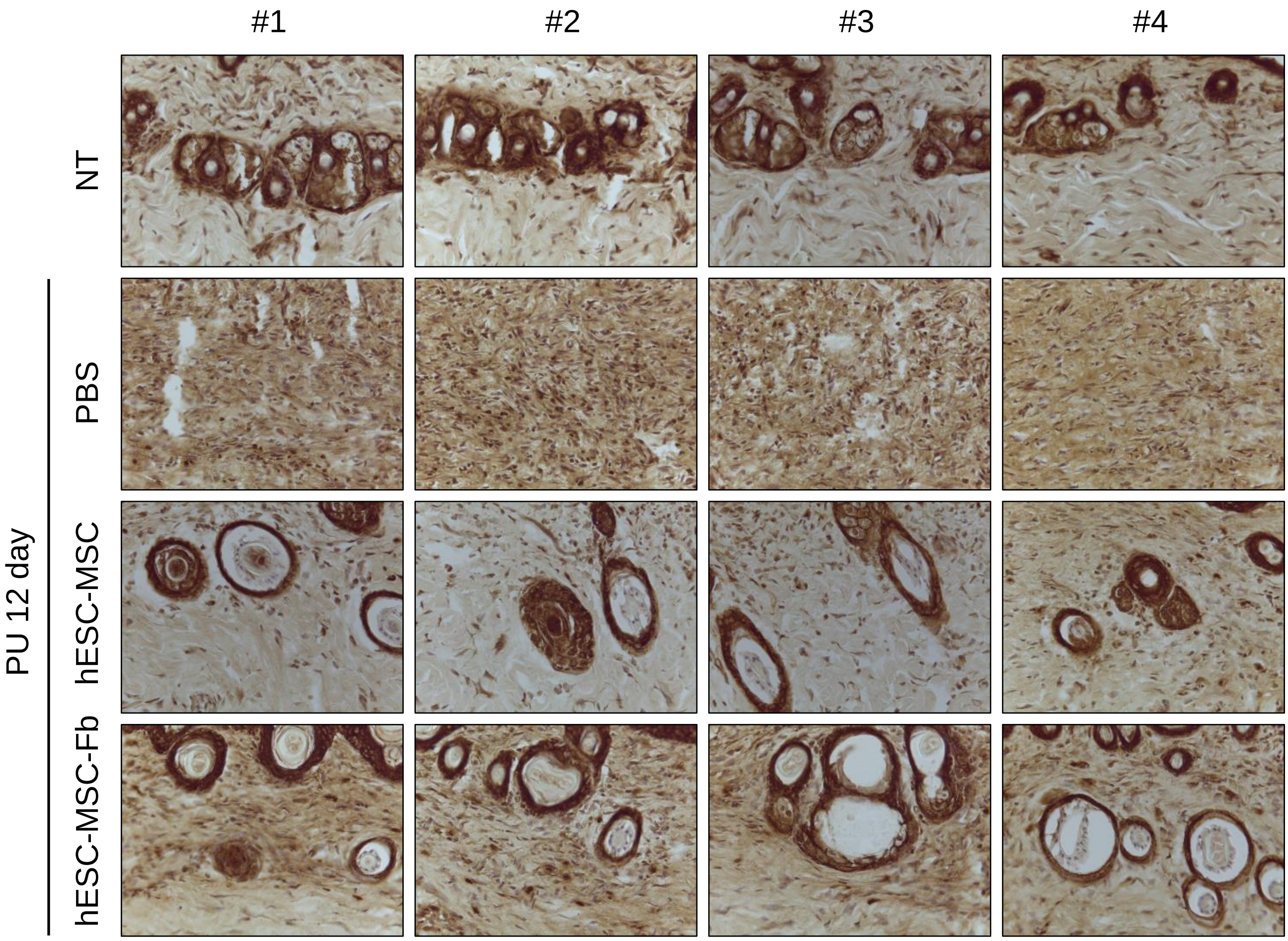

Supplement: Supplementary Materials — Supplementary Figure 1: morphological images of fibroblast differentiation of human embryonic stem cell-derived mesenchymal stem cells (hESC-MSCs) upon stimulation with connective tissue growth factor (CTGF). Supplementary Figure 2: raw data of Figures 1(a), 2(c), 4(a), 4(c), 4(d), and 4(e). Supplementary Figure 3: images of CD31 staining taken from each group for statistical analysis. [file 4789568.f1.pdf]
